# Linear Energy Transfer Incorporated Spot-Scanning Proton Arc Therapy Optimization: A Feasibility Study

**DOI:** 10.3389/fonc.2021.698537

**Published:** 2021-07-12

**Authors:** Xiaoqiang Li, Xuanfeng Ding, Weili Zheng, Gang Liu, Guillaume Janssens, Kevin Souris, Ana M. Barragán-Montero, Di Yan, Craig Stevens, Peyman Kabolizadeh

**Affiliations:** ^1^ Department of Radiation Oncology, Beaumont Health System, Royal Oak, MI, United States; ^2^ Cancer Center, Union Hospital, Tongji Medical College, Huazhong University of Science and Technology, Wuhan, China; ^3^ Advanced Technology Group, Ion Beam Applications SA, Louvain-la-Neuve, Belgium; ^4^ Center for Molecular Imaging and Experimental Radiotherapy, Institut de Recherche Expérimentale et Clinique, UCLouvain, Brussels, Belgium

**Keywords:** proton therapy, arc therapy, linear energy transfer, plan optimization, spot scanning

## Abstract

**Purpose:**

To integrate dose-averaged linear energy transfer (LET_d_) into spot-scanning proton arc therapy (SPArc) optimization and to explore its feasibility and potential clinical benefits.

**Methods:**

An open-source proton planning platform (OpenREGGUI) has been modified to incorporate LET_d_ into optimization for both SPArc and multi-beam intensity-modulated proton therapy (IMPT) treatment planning. SPArc and multi-beam IMPT plans with different beam configurations for a prostate patient were generated to investigate the feasibility of LET_d_-based optimization using SPArc in terms of spatial LET_d_ distribution and plan delivery efficiency. One liver and one brain case were studied to further evaluate the advantages of SPArc over multi-beam IMPT.

**Results:**

With similar dose distributions, the efficacy of spatially optimizing LET_d_ distributions improves with increasing number of beams. Compared with multi-beam IMPT plans, SPArc plans show substantial improvement in LET_d_ distributions while maintaining similar delivery efficiency. Specifically, for the liver case, the average LET_d_ in the GTV was increased by 124% for the SPArc plan, and only 9.6% for the 2-beam IMPT plan compared with the 2-beam non-LET_d_ optimized IMPT plan. In case of LET optimization for the brain case, the SPArc plan could effectively increase the average LET_d_ in the CTV and decrease the values in the critical structures while smaller improvement was observed in 3-beam IMPT plans.

**Conclusion:**

This work demonstrates the feasibility and significant advantages of using SPArc for LET_d_-based optimization, which could maximize the LET_d_ distribution wherever is desired inside the target and averts the high LET_d_ away from the adjacent critical organs-at-risk.

## Introduction

In the status quo of clinical proton therapy, most centers use a presumed constant relative biological effectiveness (RBE) value of 1.1 (1, 2) regardless of the dose, linear energy transfer (LET), physiological and biological factors, and clinical endpoint (3, 4). Historically, such RBE value was chosen conservatively for tumor control based on the *in vitro* and *in vivo* measurements at the center of spread-out Bragg Peak (SOBP) (2, 3). Recent experiments (5) and clinical studies (6) show that RBE value may increase towards the distal dose fall-off of SOBP as the LET increases, and this could be up to the magnitude of 1.7 (3). Hence, more than expected rate of toxicities may be seen if the distal end of the Bragg peaks ends up in the adjacent critical structures and such higher rate of toxicities, including necrosis have been reported in multiple studies (7–9). Therefore, being able to manipulate the location of high LET is very helpful to further improve modern proton beam therapy.

Nevertheless, in the era of traditional passive-scattering proton therapy (PSPT) delivery technique, the high LET region is inevitably located at the distal end of each beam and it is impossible to modulate its distribution. To avoid any overlapping high LET regions with the abutting critical organs, it is common to use the beam angles without aiming directly toward those organs. Fortunately, in the past few years, the pencil beam scanning (PBS) technique has emerged and quickly been adopted by new proton centers as the most advanced proton beam delivery technology (10, 11). Intensity-modulated proton therapy (IMPT), based on PBS, optimizes the intensities of individual spot from different energy layers. Compared with the PSPT, IMPT offers not only a more conformal radiation dose (12–14), but also has the potential to modify the LET distribution by incorporating the LET optimization into the planning process (4, 15–21). However, the ability of IMPT to spatially place the LET value is limited by the number of beam angles (22, 23) and thus not clinically feasible given the prolonged PBS treatment delivery time.

Such obstacles could be overcome by using spot-scanning proton arc therapy (SPArc) which is a novel spot scanning delivery technique that can deliver a treatment plan in an arc mode (24). SPArc has manifested significant advantages over the multi-beam IMPT (25–28) to improve dose distributions and delivery. A prototype of SPArc delivery has proven its feasibility on a state-of-the-art proton therapy system (29). Since the SPArc plans are delivered from hundreds of beam angles selected from a smart energy and spot selection algorithm (24), we hypothesize that with such increased degrees of freedom for optimization, SPArc can further improve the LET distribution while maintaining the delivery efficiency. Thus, we develop a novel LET-based SPArc optimization algorithm and explore its potential to improve the spatial LET distribution.

## Methods and Materials

### Dose-Averaged LET (LET_d_)-Based SPArc Optimization Workflow

**Figure 1:**
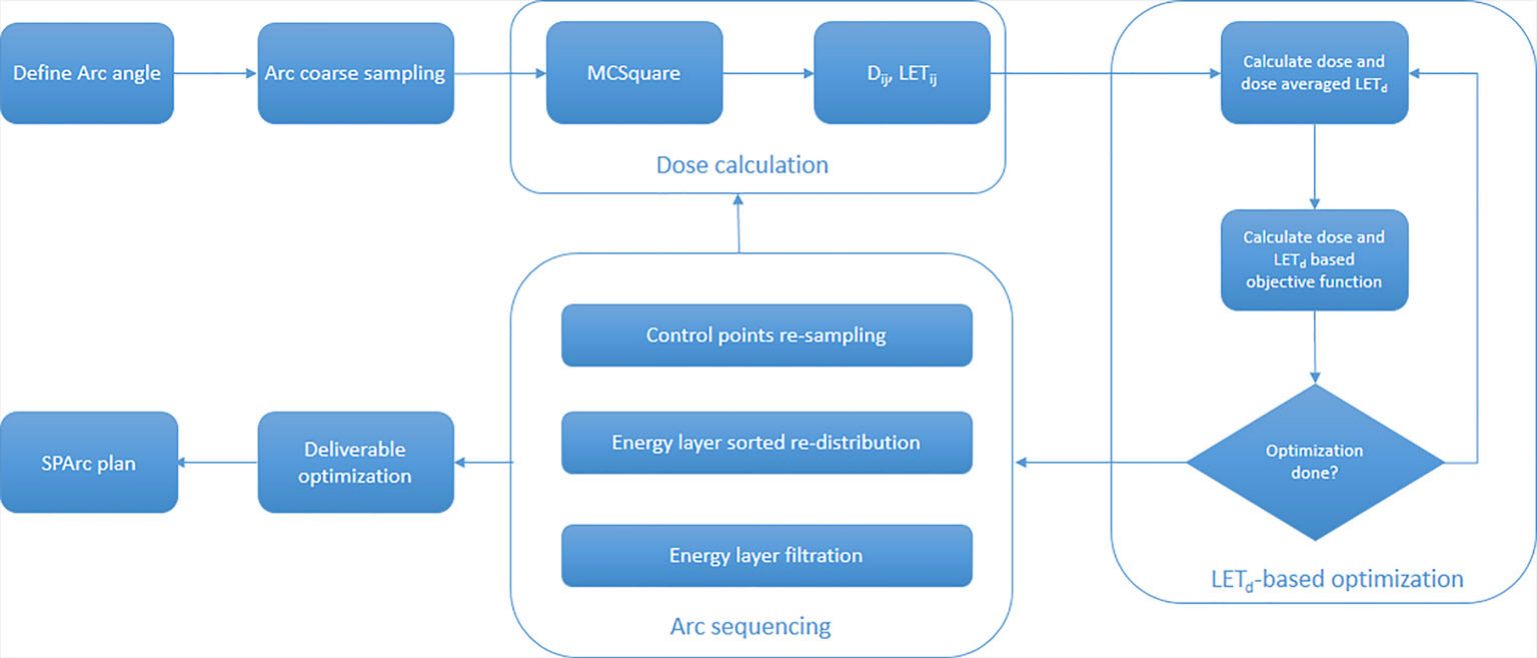
The workflow of LET_d_ based SPArc optimization engine.

The LET_d_-based SPArc optimization engine was implemented based on an open-source proton planning platform (OpenREGGUI) (30). This platform uses a fast Monte Carlo dose calculation engine (MCSquare) (31–33) and an optimizer (MIROpt) (34) for dose-based multi-beam IMPT planning. Such platform was modified to incorporate the LET_d_-based objective functions (*e.g.*, minimum and maximum LET_d_) into optimization and to iteratively generate the SPArc plans (24, 29). The details of LET_d_-based SPArc planning implementation is shown in **Figure 1** which consists of (1) the planner defines the arc start and stop angles, (2) the engine coarsely samples the initial beams (3) the MCSquare calculates the voxel dose and LET_d_ values from each spot, (4) the optimizer finds a solution by iteratively calculating the dose and LET_d_ and minimizing the dose and LET_d_ based objective functions for target volume and organs-at-risk (OARs), (5) iteratively increases the beam sampling frequency and reduces the energy layers by repeating step (3) and (4) until desired sampling frequency is reached, (6) and finally, perform deliverable optimization by specifying the minimum MU (0.02 MU) per spot. The details of the LET implementation are described as follow.

### LETd Optimization Implementation

In MCSquare calculation, the ‘stopping power’ method (35) was selected, in which LET_d_ was scored by dose-weighted average of the stopping power of particles through each voxel (36). Secondary particles were handled by specifying the options in the configuration file as: secondary photons and neutrons are not taken into account while the secondary electrons are considered as locally absorbed. Secondary protons, deuterons, and alpha’s are simulated for the dose and LET_d_. The calculated dose and LET values from each spot *j* projected to voxel *i* are expressed as (D*_ij_* and LET*_ij_*). The dose and dose-averaged LET value for voxel *i* can be calculated from the intensity (monitor unit) of the spot *j* (*ω_j_*) as follow:

(1) $$D_{i}=\Sigma_{j}D_{ij}\omega_{j}$$

(2) $$LET_{i}=\frac{\Sigma_{j}LET_{ij}D_{ij}\omega_{j}}{\Sigma_{j}D_{ij}\omega_{j}}$$

For the optimization, we used a standard quadratic objective function to control both dose and LET_d_ distributions:

(3) $$\begin{array}{c} Obj(w_{j})=Obj_{dose}(w_{j})+Obj_{LET(w_{j})}\\ =\sum_{i\in CTV}P_{CTV}{\left(D_{i}-D_{0,CTV}\right)}^{2}\\ +\sum_{i\in OARs}P_{OARs}H(D_{i}-D_{0,OARs}){\left(D_{i}-D_{0,OARs}\right)}^{2}\\ +\sum_{i\in CTV}P_{CTV,LET}H(LET_{0,CTV}-LET_{i}){\left(LET_{i}-LET_{0,CTV}\right)}^{2}\\ +\sum_{i\in OARs}P_{OARs,LET}H(LET_{i}-LET_{0,OARs}){\left(LET_{i}-LET_{0,OARs}\right)}^{2} \end{array}$$

where *p* denotes the penalty weights, D_0_ and LET_0_ correspond to the target or constraint dose and LET value respectively. The Heaviside step function *H*(D*_i_* – D_0_) is defined as convention (*i.e.*, equals to unity if D*_i_* > D_0_, otherwise equals to zero). Dose-based, dose volume based, and LET-based objective functions are implemented and used for this study.

### Treatment Planning

All the SPArc and multi-beam IMPT plans (without arc sequencing part in **Figure 1**) were generated using this optimization engine with similar parameters (*e.g.*, 10^4^ particles per spot for D*_ij_*, LET*_ij_* matrix calculation, 10^8^ particles for final dose calculation to ensure the plan is within 1% statistical uncertainties for the dose in the target volume (37), and same optimization criteria). For each patient, the IMPT plan was generated first to achieve an optimized dose distribution to the target volume and OARs without taking LET_d_ distribution into account. Based on those dose objective functions, IMPT plan was then generated by adding LET_d_ objectives of the target and OARs in the optimization. The relative weights between dose and LET_d_ objectives were further adjusted to maintain similar or superior dose distribution while maximizing and minimizing the average LET_d_ values in the target volume and OARs. In terms of SPArc planning, the plans initiated from a coarse sampling frequency of 20 degree and achieved 2.5 degree final sampling frequency using the similar objective functions to the IMPT plans with LET_d_ optimization.

### Patient Study

To quantitatively investigate the effect of the LET_d_ distribution with the relationship of beam number, a prostate patient was selected given its easy anatomical structures. A full arc SPArc and multi-beam IMPT plans with 2, 4, 6, and 8 equally spaced beams were generated to achieve similar clinical target volume (CTV, 134 cc) coverage (78 Gy in 39 fractions, using RBE of 1.1) and high dose sparing of rectum and bladder while maximizing the LET_d_ value using similar objective functions inside the CTV. Urethra was not considered as an avoidance structure for the simplicity of the analysis. However any OAR can be used as an avoidance structure in optimization although this will result in a more complicated and longer calculation process. The dose, LET_d_, corresponding dose-volume histograms (DVHs) and LET_d_-volume histograms (LVHs) of all plans were analyzed and compared with the 2-beam IMPT plan without LET_d_ optimization (2B w/o). To evaluate the treatment delivery efficiency among different plans, the delivery time was simulated based on a full gantry geometry with a rotation speed of one rotation per minute, spot switching time of 2 milliseconds, and energy-layer-switching-time of 0.6 seconds (24).

To further quantify the relationship between high LET_d_ concentration and the size of LET_d_ boost volume in SPArc optimization, different virtual simultaneously integrated boost (SIB) volumes (77.9, 39.9, 16.5, 4.5, and 0.3 ccs) were generated by shrinking the CTV at a step size of 0.5 cm. Multiple SPArc plans were generated to provide similar CTV coverage while maximizing the high LET_d_ concentrating in the SIB volumes using the similar plan parameters such as arc trajectory, sampling frequency, as well as target objective functions and weights.

A liver and a brain case were then selected to compare the resulted LET_d_ optimization between SPArc and multi-beam IMPT. For the liver case, a partial arc from 160 – 40 degree (IEC 61217) was used to prescribe 75 Gy (constant RBE of 1.1) in 25 fractions (38) to the CTV, while minimizing the mean dose to the normal liver, and maximizing the LET_d_ value in the gross tumor volume (GTV). For the brain case, the full arc was used to optimize a uniform dose to the CTV (54 Gy in 30 fractions, constant RBE of 1.1) (39) and minimize the dose to the surrounding OARs, while maximizing the LET_d_ value in CTV and minimizing the LET_d_ value to the OARs. The multi-beam IMPT plans using the clinical beam angles (two beams for the liver case, and three beams for the brain cases) were also re-optimized using the same platform with and without LET_d_ optimization. The dose, LET_d_, corresponding DVHs and LVHs were evaluated for all plans.

## Results

**Figure 2:**
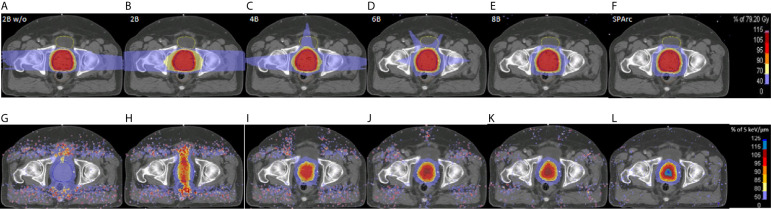
The dose (upper row) and the LET_d_ (lower row) distributions for 2 beams without LET_d_ based optimization (2Bw/o, **A, G**), and with LET_d_ based optimization for 2 (2B, **B, H**), 4 (4B, **C, I**), 6 (6B, **D, J**), 8 (8B, **E, K**) beams and SPArc **(F, L)** for a prostate case.

**Figure 3:**
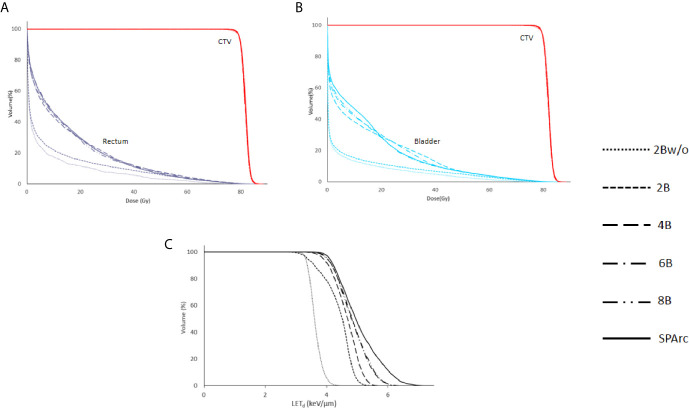
The dose volume histograms (DVHs) **(A, B)** and LET_d_ volume histograms (LVHs) **(C)** for 2Bw/o and for 2, 4, 6, 8 beams and SPArc with LET_d_ based optimization.

Dose and LET_d_ distributions for a 2-beam IMPT plan without LET_d_ optimization, and IMPT plans (2, 4, 6, 8 beam angles) and SPArc plans with LET_d_ optimization are shown in **Figure 2**. This comparison shows the power of SPArc to concentrate the high LET_d_ in the desired area of target volume. **Figures 3A, B** display the corresponding DVHs for CTV, rectum, and bladder for all plans. The corresponding LVHs for CTV are also shown in **Figure 3C**. With similar RBE 1.1 dose distributions in terms of target coverage and high dose OARs sparings, the average LET_d_ in the target increases with the number of beams used. Compared to multi-beam IMPT plans, SPArc has a better capability of spatially centralizing the LET_d_ distributions in the target while maintaining the delivery efficiency. Specifically, with LET_d_ optimization, the average value of LET_d_ in the target was 4.38, 4.65, 4.85, 4.85, and 5.06 keV/μm for IMPT plans of 2, 4, 6, 8 beams and SPArc plans, respectively. Compared with the 2-beam non-LET_d_ optimized plan, the corresponding increase of LET_d_ value was 21%, 29%, 34%, 34%, and 40% for the IMPT plans (2, 4, 6, 8 beams) and SPArc plans respectively. The estimated delivery time for the IMPT plans (2, 4, 6, 8 beams) and SPArc plans was 96, 150, 171, 188, and 125 seconds respectively. Hence, SPArc can enhance the LET_d_ distribution in the target volume more efficiently.

**Figure 4:**
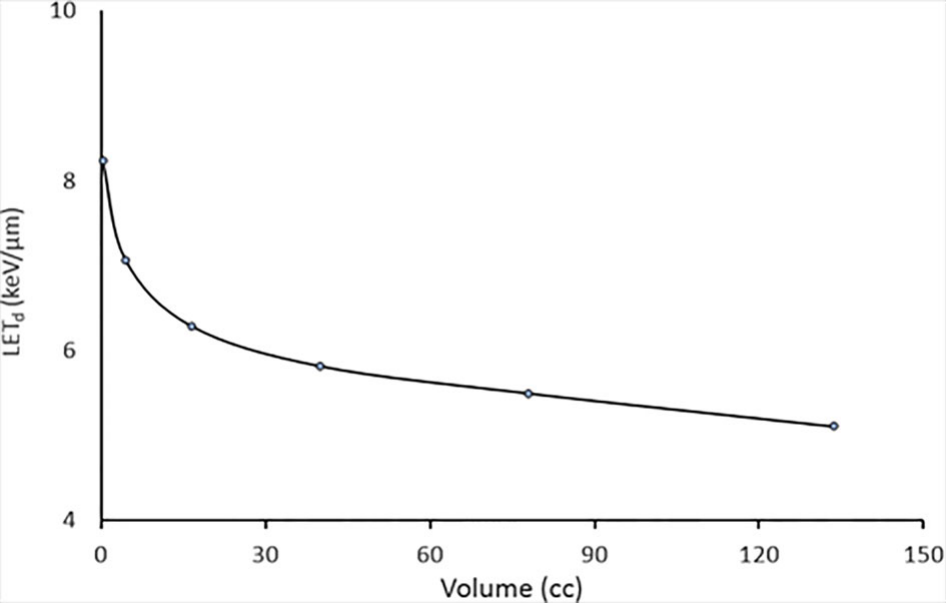
The relation with average achievable LET_d_ versus volume using SPArc while keeping similar dose to the target volume for the prostate case.

Moreover, the ability of SPArc to concentrate the LET_d_ depends on the volume as shown in **Figure 4**. The average LET_d_ value in the SIB volume for the prostate case could increase up to 8.5 keV/um as the size of the SIB volume decreases while maintaining similar dose distributions.

**Figure 5:**
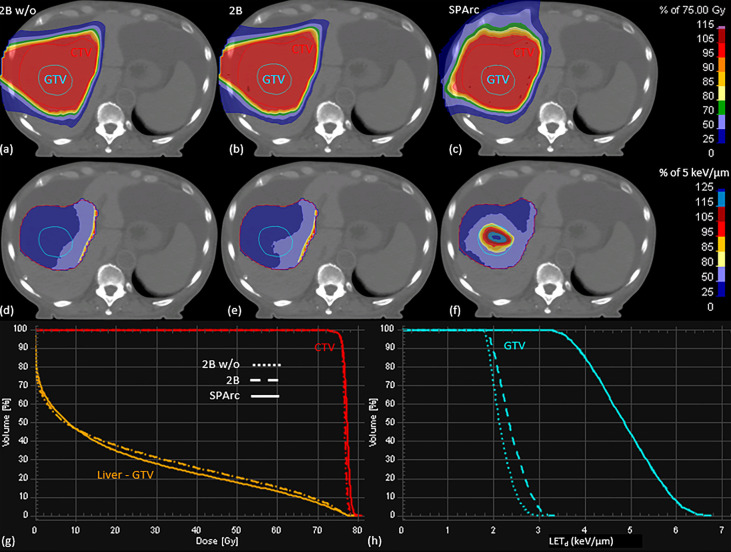
The dose (first row) and LET_d_ (second row) distributions, the DVHs **(G)** and LVHs **(H)** of 2Bw/o **(A, D)**, 2B **(B, E)**, and SPArc **(C, F)** plans for the liver patient.

To validate the capability of SPArc to optimize LET distribution, other clinical scenarios such as liver and brain cases were examined. The dose, DVHs, and LVHs for the liver case generated using two beams without LET_d_ optimization, with LET_d_ optimization, and SPArc are shown in **Figure 5**. With similar target coverage, the SPArc plans reduced the mean dose of normal liver by 1.4 Gy (RBE 1.1), and 1.5 Gy (RBE 1.1) when compared with the 2-beam IMPT plan without and with LET_d_ optimization respectively. Moreover, the SPArc could effectively escalate the high LET_d_ value in the GTV. Specifically, the average value of LET_d_ in the GTV for the liver case was 4.88 and 2.39 keV/μm for the SPArc and 2-beam IMPT plans with LET_d_ optimization respectively. Compared with the 2-beam non-LET_d_ optimized plan, the corresponding increase in LET_d_ was 124% for SPArc plan, and only 9.6% for the IMPT plan given the limited number of beam angles.

**Figure 6:**
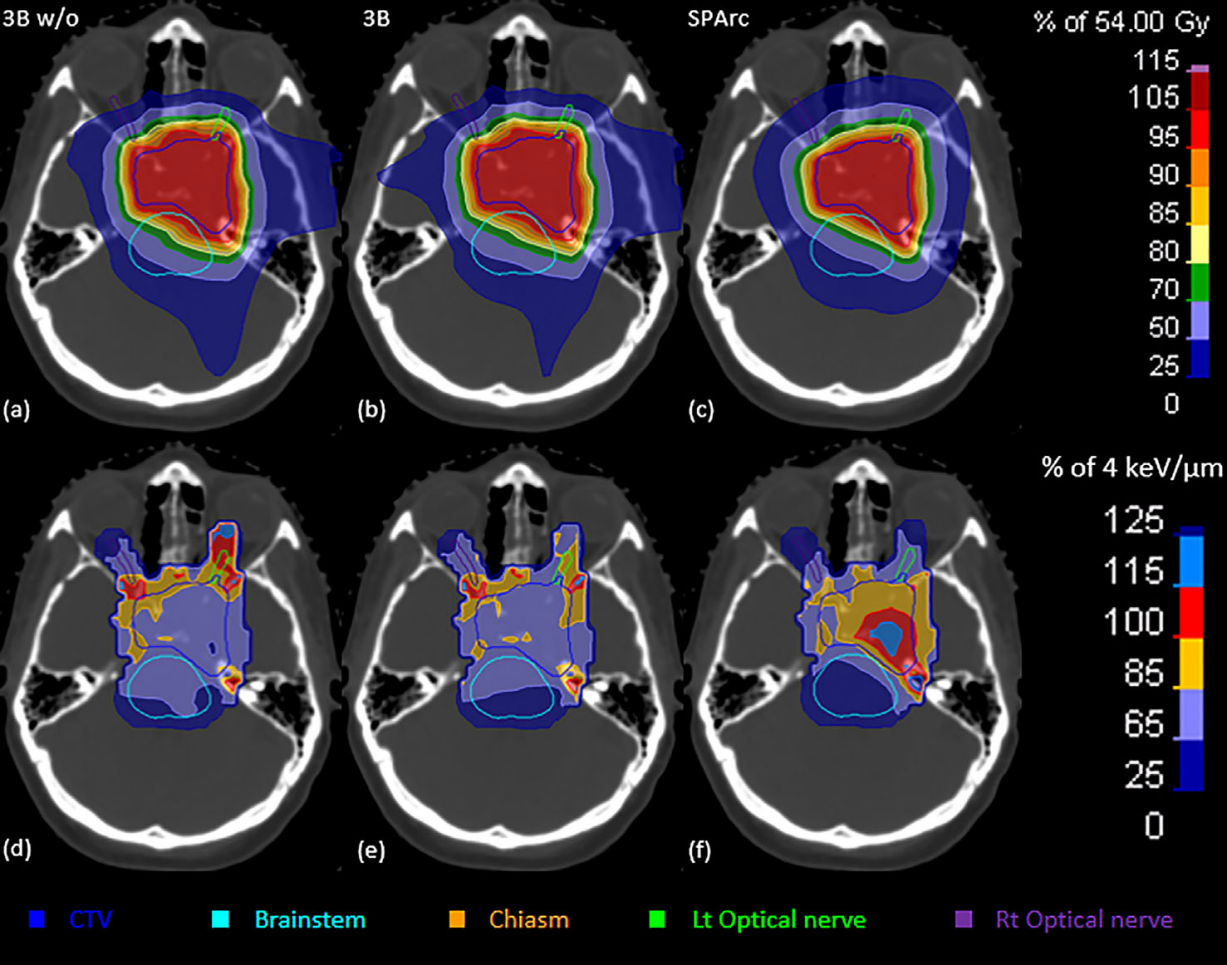
The dose (upper row) and LET_d_ (lower row) distributions of 3Bw/o **(A, D)**, 3B **(B, E)**, and SPArc **(C, F)** plans for the brain patient.

**Figure 7:**
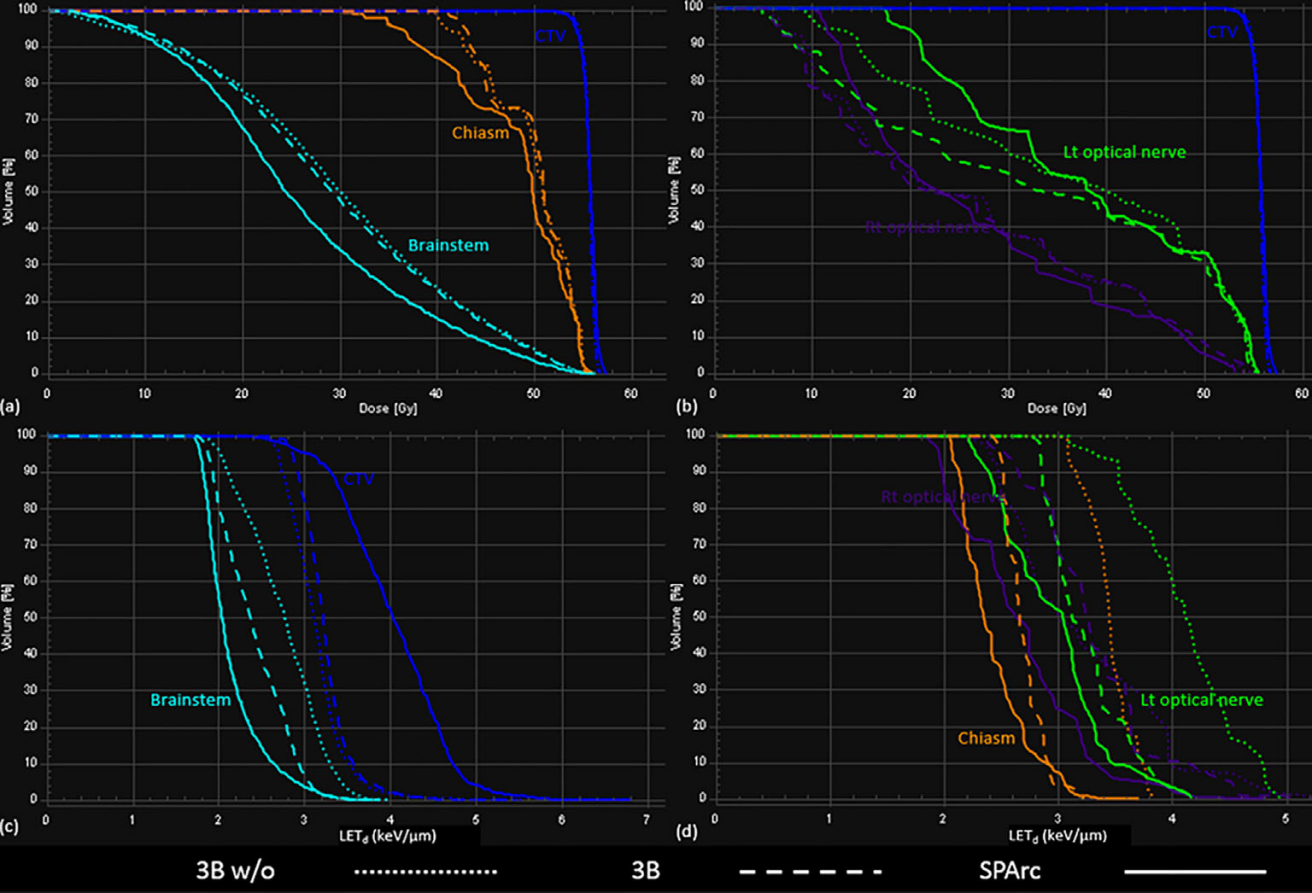
The DVHs **(A, B)** and LVHs **(C, D)** for 3Bw/o, 3B, and SPArc plans for the brain patient.

Similar results were noted in simulation of the brain case, where SPArc with LET_d_ optimization could effectively maximize the LET_d_ enhancement to the target volume while restricting the high LET_d_ away from the OARs. Specifically, the average value of LET_d_ for the SPArc plan was increased by 29% (4.03 *vs*. 3.13 keV/μm) for CTV, and was decreased by 22% (2.14 *vs*. 2.74 keV/μm), 30% (2.43 *vs*. 3.45 keV/μm), 28% (2.96 *vs*. 4.09 keV/μm), and 17% (2.66 *vs*. 3.22 keV/μm) for brainstem, chiasm, left, and right optical nerves, respectively, compared with the 3-beam non-LET_d_ optimized plan. In contrast, the corresponding improvements were only 4%, 12%, 22%, 21%, and -3% for 3-beam IMPT plan compared with the 3-beam non-LET_d_ optimized plan (**Figures 6**, **7**).

## Discussion

This is the first study to quantitatively evaluate the feasibility of SPArc to spatially optimize LET_d_ distributions using the fully LET_d_ incorporated SPArc optimization engine. Our results demonstrate that SPArc has the great capability to concentrate the high LET_d_ inside the target volume while restricting it from the OARs. Nevertheless, as the number of beam angles increases, IMPT plans may be able to achieve similar plan quality as SPArc, however this is not feasible in clinical practice due to its prolonged treatment delivery time. Several groups (40, 41) have proposed to shorten the delivery time by filtering the energy layers for IMPT plans. Such procedure is still limited and not preferable especially for multi-room proton center due to room switching time between beams (24). Conversely, SPArc has more degrees of freedom for delivering hundreds of beamlets along the arc trajectory therefore to effectively optimize the dose and LET_d_ distributions simultaneously without sacrificing the delivery efficiency.

More importantly, SPArc has demonstrated its capability of elevating the LET_d_ value inside the target. The level of elevation is dependent on the target volume (**Figure 4**). For small target volumes (*i.e.*, less than 5 cc), the average LET_d_ in the target volume could reach values higher than 7 keV/um. Compared to the average mid-SOBP LET_d_ value of 2-3 keV/um (3), the increment can be up to 250%. However, the magnitude and the conformity of LET_d_ concentration in the target volume depends on the target size, its location and more importantly on adjacent critical normal structures and their dose and LET constraints. For instance, the maximum average LET_d_ achieved in the GTV was compromised in the liver case (**Figure 5**) as only a partial arc could be used due to the location of the target. Furthermore, the irregular shape and complex geometry of the target volume and OARs can affect the distribution, conformity and the magnitude of LET_d_ in the target volume shown in the brain case (**Figure 6**) as the average LET_d_ value could only reach up to 4.03 keV/um. In order to understand the potentials and limitations of the LET_d_ distribution and its falloff *via* SPArc, more studies on different anatomical sites and patient geometries are necessary and are the mainstay of our future efforts.

This study incorporated LET_d_ optimization engine into the biological treatment planning to achieve a similar dose distribution with RBE 1.1 as used in current clinical practice. A more comprehensive biological optimization engine based on a variable RBE model could be extended from the current framework by incorporating dose, physiological and biological factors into the optimization. Thus far, none of the variable proton RBE models has been implemented into routine clinical practice due to the discrepancies between model calculations and experimental data (42, 43). In contrast to the physical parameters in the current RBE model, LET can be supported by all the variable RBE models (5), in which RBE value varies with LET values. Therefore, using this approach in this study which is maintaining similar dose (RBE 1.1) while spatially optimizing LET_d_ distribution, could be possibly clinically adopted to improve the patient outcome.

Moreover, it is important to mention that robust optimization was not incorporated into this LET_d_-based SPArc treatment planning due to the slow calculation speed and extra memory allocation. We do recognize that the dose and LET_d_ distributions could get deteriorated from the uncertainties. However, the previous study (17) have demonstrated the feasibility of improving LET_d_ distributions by integrating LET_d_ based optimization with robust optimization in IMPT plans. With the availability of better calculation models and computer hardware, we could integrate the robustness into LET_d_-based SPArc planning in our future studies.

## Conclusion

This is the first fully LET_d_ incorporated SPArc optimization algorithm and platform which has the capability to spatially optimize LET_d_. Our results demonstrate that SPArc can take advantage of arc trajectory to maximize the LET_d_ concentration to anywhere in the target volume, and to avoid the high LET_d_ from the OARs, while maintaining similar delivery efficiency. This technique will not only address one of the challenges in proton therapy which is the risk of toxicity associated with the LET uncertainty of the Bragg peak distal edge but also could provide the means to dose escalation *via* LET optimization in the target volume while sparing OARs.

## Data Availability Statement

The raw data supporting the conclusions of this article will be made available by the authors, without undue reservation.

## Ethics Statement

The studies involving human participants were reviewed and approved by Beaumont Health Institutional Review Board. Written informed consent for participation was not required for this study in accordance with the national legislation and the institutional requirements.

## Author Contributions

XL designed the study, performed the data acquisition and analysis, wrote the manuscript. XD, WZ, and GL assisted in designing the study and revising the manuscript. GJ, KS, and AB-M provided intensive feedback to improve the algorithm. DY and CS provided physics and clinical support. PK helped to design the study, provided clinical inputs, and revised the manuscript. All authors contributed to the article and approved the submitted version.

## Funding

XD and XL received the fund from Ion Beam Applications. The funder was not involved in the study design, collection, analysis, interpretation of data, the writing of this article or the decision to submit it for publication.

## Conflict of Interest

XD, XL, and DY have a patent related to the Particle Arc Therapy (WO2017156419). GJ was employed by Ion Beam Applications SA.

The remaining authors declare that the research was conducted in the absence of any commercial or financial relationships that could be construed as a potential conflict of interest.

## Abbreviations

LET_d_, dose-averaged linear energy transfer; SPArc, spot-scanning proton arc therapy; MPT, intensity-modulated proton therapy; RBE, relative biological effectiveness; SOBP, spread-out Bragg Peak; SPT, passive-scattering proton therapy; PBS, pencil beam scanning; OARs, organs-at-risk; DVH, dose-volume histogram; LVH, LET_d_-volume histogram; 2B w/o, 2-beam IMPT plan without LET_d_ optimization; SIB, simultaneously integrated boost; GTV, gross tumor volume.
